# Evolution of astrocytes: From invertebrates to vertebrates

**DOI:** 10.3389/fcell.2022.931311

**Published:** 2022-08-15

**Authors:** Carmen Falcone

**Affiliations:** Department of Neuroscience, International School for Advanced Studies (SISSA), Trieste, Italy

**Keywords:** astrocyte, evolution, central nervous system, glia, vertebrates, astrocytes

## Abstract

The central nervous system (CNS) shows incredible diversity across evolution at the anatomical, cellular, molecular, and functional levels. Over the past decades, neuronal cell number and heterogeneity, together with differences in the number and types of neuro-active substances, axonal conduction, velocity, and modes of synaptic transmission, have been rigorously investigated in comparative neuroscience studies. However, astrocytes, a specific type of glial cell in the CNS, play pivotal roles in regulating these features and thus are crucial for the brain’s development and evolution. While special attention has been paid to mammalian astrocytes, we still do not have a clear definition of what an astrocyte is from a broader evolutionary perspective, and there are very few studies on astroglia-like structures across all vertebrates. Here, I elucidate what we know thus far about astrocytes and astrocyte-like cells across vertebrates. This information expands our understanding of how astrocytes evolved to become more complex and extremely specialized cells in mammals and how they are relevant to the structure and function of the vertebrate brain.

## Introduction

The central nervous system (CNS) shows incredible diversity across evolution at the anatomical, cellular, molecular, and functional levels. Over the past decades, the number and heterogeneity of neuronal cells, together with differences in the number and types of neuro-active substances, axonal conduction, velocity, and modes of synaptic transmission, have been among the most investigated characteristics in comparative studies of the brain.

**FIGURE 1:**
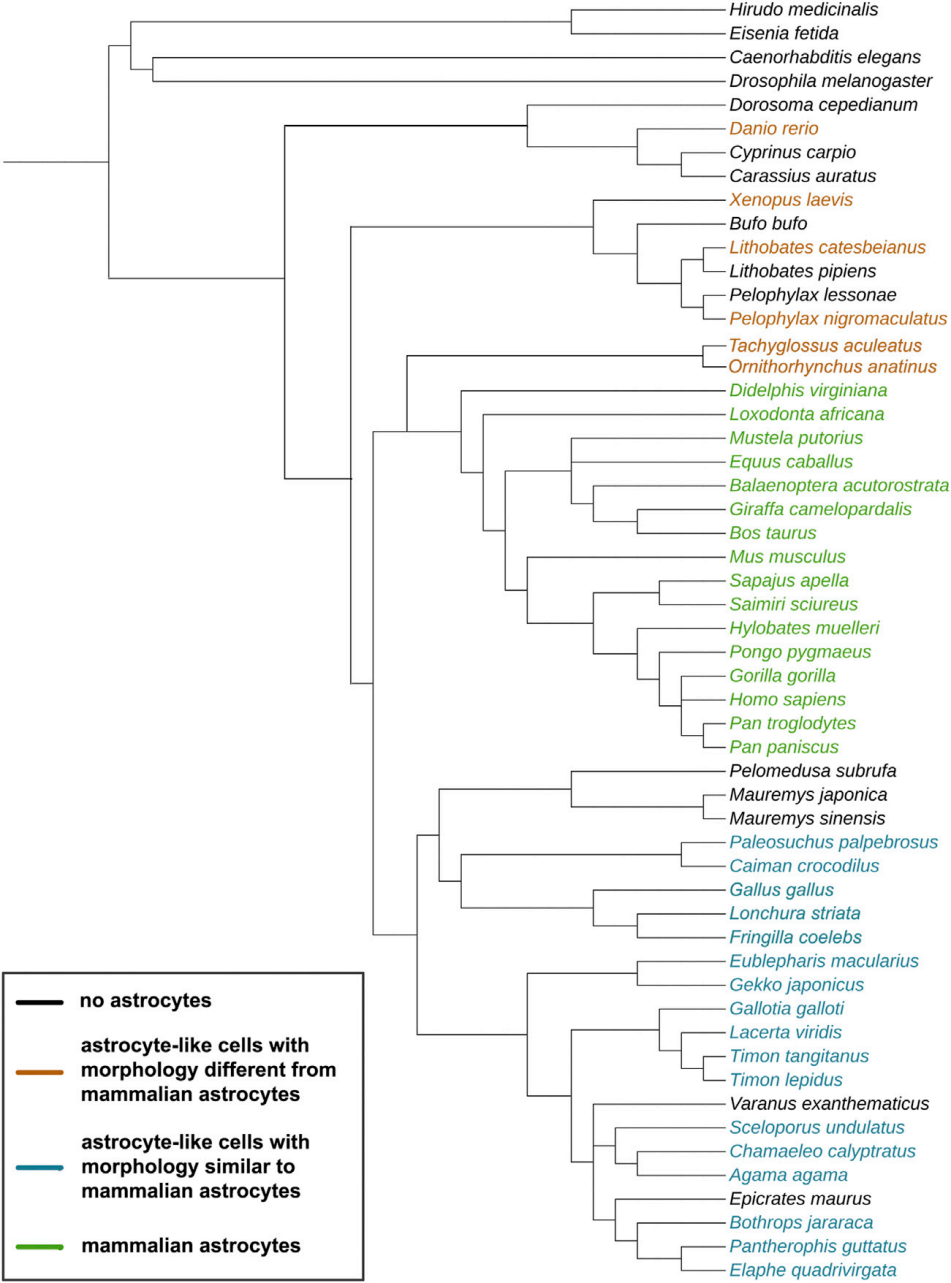
Evolutionary tree of the species discussed in this study. The cladogram was created on https://phylot.biobyte.de/, based on the NCBI taxonomy database. Color code: black = no astrocytes; orange = species showing astrocyte-like cells with morphology different from mammalian astrocytes; blue = species showing astrocyte-like cells with morphology similar to mammalian astrocytes; green = species showing mammalian astrocyte morphology.

Astrocytes, a specific type of glial cell in the CNS, play pivotal roles in regulating all of these features and thus are crucial for the development and evolution of the CNS. However, they have received less attention in comparative studies. Though special attention has been paid to mammalian astrocytes, we still do not have a clear definition of what an astrocyte is from a broader evolutionary perspective, and very little research has been done on astroglia-like cells across different non-mammalian species. The purpose of this review is to report what we do know about astrocytes and astrocyte-like elements across vertebrates and about primitive astroglia-like structures in invertebrates (see Figure 1 for the species included in this review). This analysis will help us understand how astrocytes evolved to become more complex and extremely specialized cells in mammals and how they are relevant to the structure and function of the brain in vertebrates.

### Astrocyte identity and the origin of neuroglia

The term “astrocytes” was used for the first time by Lenhossek in 1895 (Lenhossék M. 1895) with the intent to replace the term “glia” (from the Greek for “glue”), which did not represent the many functions of these cells. Lenhossek based his term on the cell’s morphology and, specifically, on the presence of multiple cellular processes resembling those of a star. The first nomenclature by Del Río Hortega (Del Rio-Hortega P. 1932; Hortega 1942) distinguished two types of astrocytes in mammals—protoplasmic and fibrous astrocytes—based on their morphology and position in the CNS. These are still considered the main types of astrocytes; however, already in 1965, Duncan (Duncan D. 1965) noted that this classification was an oversimplification of astrocyte heterogeneity.

Astrocytes are indeed a highly heterogeneous cell population, with different morphologies, molecular profiles, distributions in different anatomical regions, physiology, and functions across different species. The fact that there is still no unique marker to label all astrocytes and not other brain cell types makes it challenging to define criteria for classifying astrocytes across different species. However, several attempts have been made to describe astroglia across nervous systems of different species and to identify primitive astrocyte forms in more ancient vertebrate and invertebrate species.

Astrocytes have been broadly described in mammals, where they have reached their highest specialization. Currently, astrocytes are known to play crucial roles in the CNS, such as regulating water and ion homeostasis and exchanging nutrients across the blood-brain barrier. They are also pivotal players in the development and regulation of connectivity, such as in synapse formation and pruning, and in synaptic function and plasticity across development and in the adult. Moreover, astrocytes are able to react to injury or stress with a series of processes called reactive astrogliosis, which results in scar formation or glial borders (Sofroniew 2009; Sofroniew and Vinters 2010; Sofroniew 2015; 2015b) and thus exerts both a protective and neurotoxic effect (Escartin et al., 2021). Astrocytes likely co-evolved with neurons and became more and more specialized in mammals compared to other vertebrates due to mammals’ higher CNS complexity and higher energy demands. Understanding how astrocytes evolved across species is crucial to understanding their contribution to CNS complexity and functions in human and non-human primates.

When we look at the most ancient nervous systems in invertebrates, only neurons—which evolved from epithelial cells—are present, and they are organized in a diffuse network. The nervous system showed its first organization in neuronal ganglia in Cnidaria, then became a proper centralized CNS in Bilateria (e.g., flatworms), subsequently became more complex in protostomes (e.g., insects and crustaceans), and finally showed higher degrees of complexity in vertebrates. The appearance of a CNS necessitated supportive cells, i.e., glial cells (Verkhratsky, Ho, and Parpura 2019). The specific phylogenetic relationships of glial cells between invertebrates and vertebrates are still debated. In fact, different studies have shown that different genes are involved in glial differentiation in invertebrates vs vertebrates (e.g., gcm drives glial differentiation in *Drosophila*, while the mammalian homologue Gcm has not retained that function) and that there are highly conserved pathways between the two groups (e.g., BMP FGF) (Yang and Jackson 2019).

**FIGURE 2:**
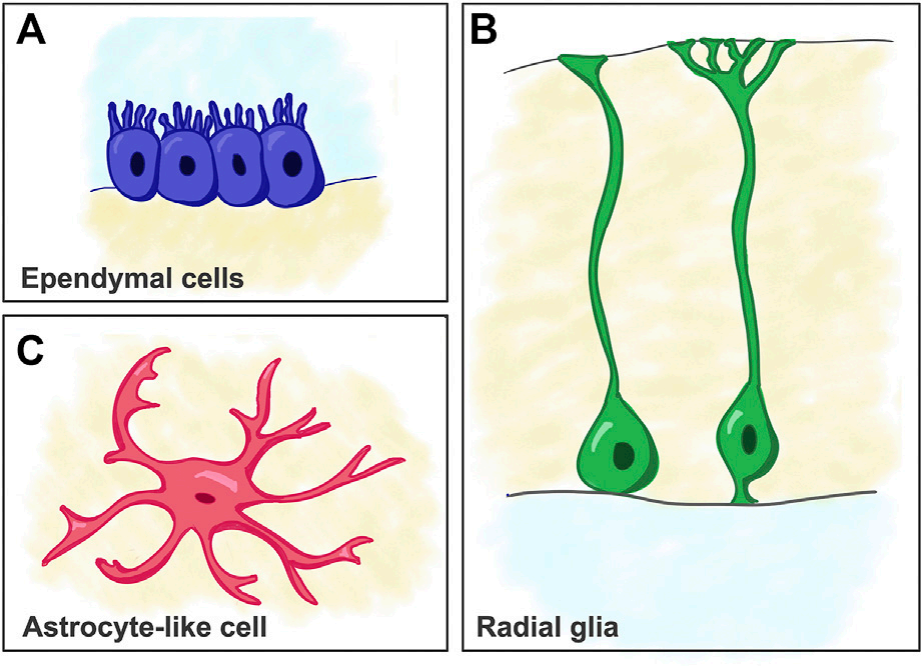
Schematic of cell types considered as predecessors of mammalian astrocytes. **(A)** Ependymal cells. **(B)** Radial glia cells. **(C)** Astrocyte-like cells (e.g. as seen in reptiles).

The first glial cells are visible in Acoelomorpha, although they became more complex (e.g., sheath glia) in *Caenorhabditis elegans* and Anellida, with anatomical and functional features of both mammalian astrocytes and oligodendrocytes. In Deuterostomes, radial glia (RG) cells are seen for the first time, then in early Chordata, they became the predominant type of glial cells and are present throughout life (Figure 2B). RG cells show a characteristic radial morphology with an elongated shape of the cell bodies, long radial processes directed into the parenchyma, and the typical expression of intermediate filaments in the cytoplasm. RG cells represent the main type of glia in the adult CNS of many early vertebrates, where they exert functions similar to those of mammalian astrocytes. For example, the well-developed RG cells in zebrafish send processes travelling from the ventricles to the pial surface, show robust glial fibrillary acidic protein (GFAP) expression, and are involved in glutamate and water homeostasis (thanks to *Gs* and *Aqp4* expression). However, RG cells are considered purely neural stem cells in the mammalian CNS, where they are abundant during embryonic development but largely disappear after birth. Across evolution, we witness an increase in the complexity of the CNS, accompanied by a need for the specialization and diversification of neuroglia, and more specifically, of astrocytes. Astrocyte complexity and heterogeneity is especially noticeable in primates (Verkhratsky, Ho, and Parpura 2019).

### Methods for conducting comparative studies of astrocytes

Due to the lack a comprehensive description of what an astrocyte is across species and due to astrocytes’ tremendous heterogeneity, to identify their presence, we often rely on the astrocytic features that we find in mammals, the group in which these cells have been most frequently investigated. However, astrocytes or astrocyte-like cells can be studied in different species comparatively with regard to morphology, gene expression, and functions under normal conditions and in reaction to injury or stress.

Some pivotal studies comparing the presence of astrocytes across different species are very old and are mostly based on morphology; nevertheless, they still convey fundamental information (some of these pivotal morphological studies are reviewed in (King 1966). However, those studies need to be followed up with research that employs more modern technologies. For example, most evidence of astrocytes’ presence across different vertebrates relies on immunohistochemistry against GFAP, a type III intermediate filament protein. Morphological observations include cell shape and position; shape; number and direction of processes; contact with blood vessels, neurons, and synapses; and cellular distribution in different regions of the CNS. The GFAP gene has been shown to be highly conserved in vertebrates and has been considered for decades as the main marker for astrocytes; however, it has been recognized to label only a subpopulation of astrocytes and to label also other cell types such as RG cells. Therefore, though GFAP expression, when combined with anatomical and structural observations, is still valid for identifying astrocyte-like cells across many different species, it may be insufficient to comprehensively define the features and heterogeneity of the whole astrocyte population. For example, *tiling* (a process for which astrocytes cover discrete, non-overlapping anatomical territories in the parenchyma) has never been tested in all astrocytes due to the use of only GFAP and S100b markers (O’Leary and Mechawar 2021). In the future, the use of multiple astrocyte markers will be fundamental for analyzing specific astrocyte features.

Criteria for identifying astrocytes and astrocyte-like cells across evolution should, however, not be limited to the study of their morphology, but also of their physiological properties and their molecular profiles. More recent studies have taken advantage of the RNA expression of genes involved in astrocyte differentiation and function across species belonging to vertebrate and invertebrate species (Chen et al., 2020; Li et al., 2021). However, such studies are still few and do not include broad collections of species across evolution. The most relevant functional studies of astrocytes across species involve the comparison of calcium (Ca^2+^) signaling (intracellular Ca^2+^ elevations, with specific spatial and temporal properties, involved in the regulation of synapses, circuits, and ultimately, behavior) and gliotransmission (Guerra-Gomes et al., 2018). A few studies have also looked at how astrocytes react after injury in different species, with a special focus on astrocytes’ reactivity after traumatic brain or spinal cord injury (Gu et al., 2015; Du et al., 2021; Perez, Gerber, and Perrin 2021).

Here, I will review both old and recent findings of astrocytes and astrocyte-like cells across evolution.

### Astroglia in invertebrates

Among the most relevant challenges in comparative studies of glia between vertebrates and invertebrates is the almost total absence of markers conserved between the two groups. Early invertebrates present primitive neuroglia that likely represent the predecessor of vertebrate astroglia. Flatworms are the first Bilateria presenting a centralized nervous system: they show glia-like mesenchymal cells exhibiting long processes that contact nervous cells. Earthworms have slightly more morphologically complex neuroglia that show, for the first time, a certain degree of specialization. In fact, earthworm (*Eisenia fetida*) neuroglia include different types of cells: neurilemmal, subneurilemmal, supporting-nutrifying, and periaxonal sheath-forming cells. Among these, the supporting-nutrifying neuroglia are GFAP-immunoreactive and play roles similar to the nutritive roles of mammalian astrocytes (Coles 2009; Csoknya, Dénes, and Wilhelm 2012; Verkhratsky, Ho, and Parpura 2019). The nematode *C. elegans* presents what have been defined as “proto-astrocytes”—a type of neuroglia involved in several functions similar to those of mammalian astrocytes, but mostly associated with sensory systems. Among the different types of neuroglia in *C. elegans*, the cephalic sheath cells in the nerve ring control ion homeostasis in perisynaptic regions and are involved in regulating neuronal development and morphogenesis and in suppressing locomotion during sleep (Bacaj et al., 2008; Oikonomou and Shaham 2011; Stout Jr; Katz et al., 2018; Verkhratsky et al., 2018). However, both glial and neuronal cells are different from the neuroglia of other species in terms of morphology and physiology. For example, due to the lack of a circulatory system in *C. elegans*, such proto-astrocytes do not form the glia limitans barrier that we see in mammals nor do they express mammalian glia markers. Members of the phylum Anellida have *homeostatic* proto-astrocytes. The leech (*Hirudinea medicinalis*)’s glial cells are interconnected due to the presence of gap junctions. In this species, *packet glial cells* are able to buffer extracellular K^+^, and *giant glial cells* exhibit processes that contact neuronal dendrites (Nicholls and Kuffler 1964; Munsch and Deitmer 1992; Saubermann, Castiglia, and Foster 1992). In insects—and more specifically in the common fruit fly (*Drosophila melanogaster*), a model organism studied extensively in neuroscience—proto-astrocytes are present; they not only display a higher degree of specialization (compared to other invertebrates) but also are analogous to typical astrocytes in terms of physiology and function. The *Drosophila surface glia* are in charge of building a brain-hemolymph barrier, resembling what would later evolve into the blood-brain barrier in vertebrates. The *cortex glia* have processes contacting neuronal cell somata, with each glial cell contacting multiple neurons, and display calcium oscillations similar to those occurring in mammalian astrocytes. The *neuropil glia* include ensheathing/fibrous and astrocyte-like glia. Like mammalian astrocytes, the *Drosophila* astrocyte-like glial cells in the neuropil wrap axons and synapses (i.e., one astrocyte-like cell contacts many neuronal synapses) and are implicated in synaptogenesis and synaptic transmission. Interestingly, such astrocyte-like cells show many arborizations in their processes for the first time, thus they are quite similar to vertebrate astrocytes from a morphological perspective (Freeman and Doherty 2006; Parker and Auld 2006; Edwards and Meinertzhagen 2010; Hartenstein 2011) (Figure 2C). In addition, these cells also employ Ca^2+^-dependent signaling mechanisms, they are electrically coupled by gap junctions, and they show tiling, in which astrocytes cover discrete, non-overlapping anatomical territories in the parenchyma. Genomic studies in *Drosophila* have revealed genes important in glial differentiation and functions. *Glial-cell missing* (*gcm*) is one of the most important genes regulating glial lineage differentiation in the fly, but those same functions in glial development have not been preserved in its homologue in mammals (*Gcm1*). However, comparative studies between fly, mouse, and human gene transcription have shown that there is a certain degree of gene conservation that points to a common evolutionary origin of glial cells among invertebrates and vertebrates. Among the 900 genes that are orthologues among these species, there are transcription factors, ion channels, and transporters important in mammalian astrocyte physiology (Yang and Jackson 2019). For example, *Drosophila* astrocyte-like glia express GABA transaminase (*Gat-1*), the dEAAT1 glutamate transporter, and glutamate synthase 2 (*Gs2*) (Yang and Jackson 2019). Glial cells in the fly are also critical in regulating circadian rhythms, a function that is conserved in astrocytes of mouse suprachiasmatic nuclei (Brancaccio et al., 2017, 2019; Yang and Jackson 2019).

### Astroglia in fish

A series of morphological studies have been conducted in both model and non-model organisms of teleosts, which comprise the largest number of fish species, widely distributed in different habitats. In one carp species (*Cyprinus auratus*), mostly ependymal glia were found, with processes not contacting blood vessels (Achúcarro N. 1915; King 1966) (Figure 2A). In another species of carp, *Cyprinus carpio*, GFAP expression has been studied in depth (Onteniente, Kimura, and Maeda 1983). The authors found dense GFAP immunoreactivity within thick and straight bundles of processes around ventricles in the adult, both in sub-ependymal areas and in strict association with blood vessels. Strong GFAP expression has also been documented in white matter (especially in the optic tract and in the fasciculus longitudinalis medialis), while it has not been found in grey matter, with the exception of the optic tectum and mesencephalon, where rare radial processes end on the pial surface with small endfeet. Again, no proper astrocytes have been observed in this species of carp (Onteniente, Kimura, and Maeda 1983).

In the American gizzard shad (*Dorosoma cepedianum*), ependymal cells are still the predominant type of glia; however, they show some similarities with astrocytes in terms of process endings. In fact, their processes contact neurons and blood vessels and thicken beneath the pial surface. Moreover, there are both bipolar and tripolar non-ependymal cells found close to large neurons, without typical perivascular endfeet. It has been suggested that these types of non-neuronal cells could be predecessors of astrocytes and oligodendrocytes as we know them in mammals (King 1966).

Astroglia-like cells have been investigated in depth in the well-studied animal model zebrafish (*Danio rerio*) in a pivotal paper recently published by (Chen et al., 2020). While previous studies proposed that RG would functionally substitute for astrocytes in the adult zebrafish nervous system, Chen et al. showed that zebrafish spinal cord RG differentiate into cells that share several similarities with mammalian astrocytes. They found that such spinal cord RG cells express mammalian astrocyte marker genes such as Glt-1, Glast, and Gat-3 across development. With elegant genetic manipulations and *in vivo* imaging, they were able to document the transformation from RG to astrocyte-like cells—a transformation that revealed dynamic cellular process elaboration and arborization early in development (between two and 4 days post fertilization). These astrocyte-like cells express glutamine synthetase in somata and processes, and their processes are closely associated with synapses (labeled with the synaptic vesicle glycoprotein 2A, SV2). Interestingly, these cells are able to establish individual cell territories with minimum overlap with each another, similar to what mammalian astrocytes do with their tiling. This work is also among the few to show similarities in electrophysiological kinetics between zebrafish astrocyte-like cells and mouse astrocytes. Zebrafish astrocyte-like cells present spontaneous microdomain Ca^2+^ transients in their fine processes, and, more specifically, respond to norepinephrine activation, thus displaying calcium dynamics similar to awake behaving mice. Finally, this *bona fide* astrocytic cell population shows a conservation of factors involved in astrocyte morphogenesis, such as Fgfr3 and Fgfr4. These findings are not only important from an evolutionary perspective but also point to the zebrafish as a valuable model for investigating the molecular mechanisms that govern astrocyte functions (Chen et al., 2020).

### Astroglia in amphibians

Very few studies on glial cell identity and functions have been conducted in amphibians. In the frog *Rana esculenta*, an RG morphology similar to that of fish has been observed, though with thicker processes contacting blood vessels (similar to mammalian RG and astrocyte endfeet). However, both in the frog *Pelophylax esculentus* and in the toad *Bufo vulgaria*, ependymal cells remain the predominant type of glial cell, forming a sparse glial network across the CNS (Achúcarro N. 1915; Bairati and Maccagnani 1950; King 1966). Anatomical studies done specifically in the frog *Lithobates pipiens* reported two densely packed rows of ependymal cells in the primitive hippocampus and striatum, sending out processes that travel through the superficial neuronal layers and course very close to the neuron somata, sometimes contacting them. Moreover, very few non-ependymal cells with processes in close association with neurons have been observed in the same frog species (King 1966). In the frog species *Lithobates catesbeianus* and *Pelophylax nigromaculatus*, astrocyte-like cells have been observed, but, like in other amphibians, no typical stellate astrocytes have been detected (Onteniente, Kimura, and Maeda 1983). In the frog animal model *Xenopus laevis*, astrocyte-like glial cells express Blbp, a well-known marker for RG in mammals, thus they show a molecular expression profile that more closely resembles that of immature mammalian astrocytes or RG (Mills et al., 2015).

### Astroglia in reptiles

**FIGURE 3:**
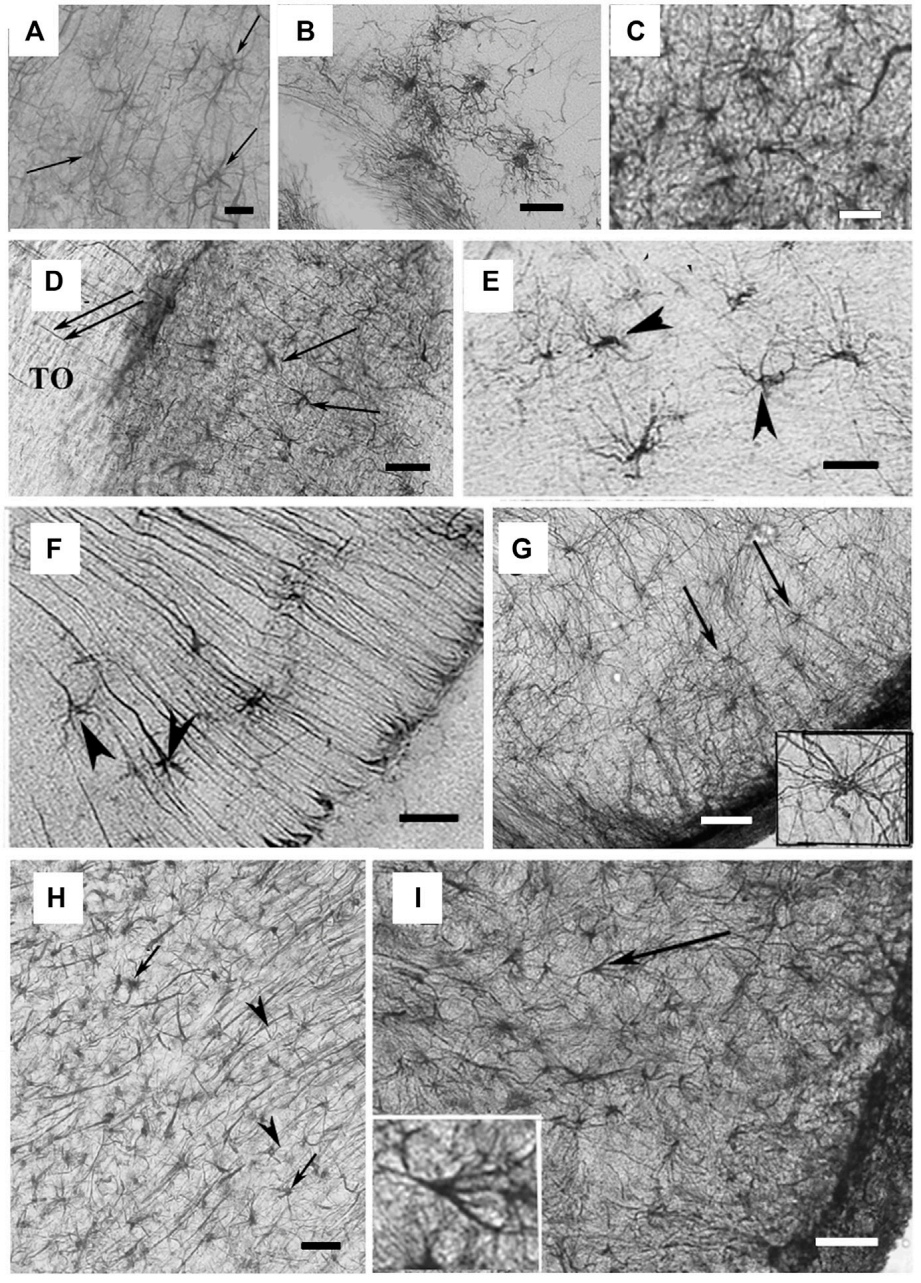
Astrocyte-like cells in reptile brains. **(A)** Adapted from Lõrincz and Kálmán, 2020, from Figure 4H. GFAP-immunopositive elements in agama telencephalon; arrows point to astrocytes intermingled within RG processes in the nucleus accumbens. Scale bar: 20 µm. **(B)** Adapted from Lõrincz and Kálmán, 2020, from Figure 5F. Astrocytes from the chameleon septum. Scale bar: 20 µm. **(C)** Adapted from Lõrincz and Kálmán, 2020, from Figure 5G. Astrocytes from the chameleon hypothalamus. Scale bar: 20 µm. **(D)** Adapted from Lõrincz and Kálmán, 2020, from Figure 8C. Arrows point to astrocytes within the optic tract (TO) of Moroccan eyed lizard diencephalon. Scale bar: 80 µm. **(E)** Adapted from Lõrincz and Kálmán, 2020, from Figure 9J. Arrowheads point to astrocytes in the chameleon brain. Scale bar: 20 µm. **(F)** Arrowheads point to astrocytes soma in the chameleon brain. Scale bar: 20 µm. **(G)** Adapted from Lõrincz and Kálmán, 2020, from Figure 13J. Arrows point to astrocytes with long processes in the brain stem of the python. Scale bar: 50 µm. **(H)** Adapted from Lõrincz and Kálmán, 2020, from Figure 10G. Astrocytes in the corn snake brain; arrows point to astrocytes, arrowheads point to RG processes. Scale bar: 40 µm. **(I)** Adapted from Lõrincz and Kálmán, 2020, from Figure 13L. Astrocytes in the ventrolateral part of the brain stem in the corn snake; arrow points to a cell enlarged in the inset. Scale bar: 50 µm.

Reptiles represent a key group in the phylogenetic evolution of astroglial cells because they are the first to that show cells with clear astrocyte morphology (Bodega et al., 1990). Several studies have investigated the presence of astrocytes in reptiles and have shed light on the heterogeneity of their distribution and on putative astrocyte predecessor cells in multiple brain regions. In general, in the reptile forebrain, the predominant cells are unipolar ependymal cells and bipolar RG-like cells with processes directed to the parenchyma, with sparse astrocytes close to neurons and almost always intermingled within RG fibers (Bairati and Tripoli 1954; King 1966). Drs. Lõrincz and Kálmán reviewed the most relevant findings in Squamata, the largest order of reptiles comprising lizards and snakes (Lõrincz and Kálmán 2020). In the telencephalon and anterior hypothalamus of the leopard gecko (*Eublepharis macularius*), GFAP^+^ RG cells are organized in a layered structure with a lighter middle zone less densely packed with GFAP-immunoreactive fibers and dense with neurons. In the septum, lateral pallium, and dorsal ventricular ridge (DVR), the glial structure is more complex, but is almost devoid of GFAP expression. In the same species, GFAP is instead evenly distributed in the diencephalon. Very few astrocyte-like cells have been observed in the ventricular surface of the mesencephalon, and, more specifically, in the torus semicircularis (Lõrincz and Kálmán 2020). In the monitor lizard (*Varanus exanthematicus*), the telencephalon shows strong GFAP expression, with a trilaminar structure like in the gecko, while the DVR shows little to no GFAP immunoreactivity (Lõrincz and Kálmán 2020). In agama (*Agama*), the telencephalon is low in GFAP expression, with GFAP being detected mostly in the mediodorsal pallium, septum, striatum, and amygdala. Few cells with typical astrocyte morphology are visible in the septum and nucleus accumbens, and they are intermingled among RG fibers (Lõrincz and Kálmán 2020) (Figure 3A). In the agama diencephalon, GFAP has a variable distribution, with RG fibers penetrating the thalamus and hypothalamus (Lõrincz and Kálmán 2020). Similarly, in the chameleon (*Chamaeleo calyptratus*), GFAP^+^ cells are present in the medial pallium and the septum, with the striatum penetrated by arching RG processes, while they are mostly absent in the diencephalon and the DVR. GFAP^+^ stellate astrocytes are present in both the septum, the preoptic hypothalamus, and, in small groups, in the tegmentum. In the agama optic tract and spinal cord, astrocytes are intermingled within RG fibers (Lõrincz and Kálmán 2020) (Figures 3B,E,F). In the lacertid Moroccan eyed lizard (*Timon tangitanus*), there is an intermediate distribution of GFAP immunoreactivity compared to the lizard species discussed above. Most of the DVR is deficient in GFAP^+^ cells or fibers. In the telencephalon, glial processes terminate on vessels with wide, round endfeet, similar to mammalian astrocytes. Here, the diencephalon presents a similar structure to other lizards, with the optic tract showing a zone dense with astrocytes (Lõrincz and Kálmán 2020) (Figure 3D). The Moroccan eyed lizard is the only lizard to have astrocytes in the mesencephalon (Lõrincz and Kálmán 2020). In the European green lizard (*Lacerta viridis*), ependymal cells show pial ending, while non-ependymal neuroglia are present in the basal ganglia and the septal nucleus (Achúcarro N. 1915; King 1966). In the eastern fence lizard (*Sceloporus undulatus*), ependymal cells with single elongated processes are detectable in the primitive hippocampus and striatum, while non-ependymal glial cells show a small, bipolar morphology or a large, oval, multipolar shape similar to that of mammalian astrocytes (King 1966). In the Western Canaries lizard (*Gallotia galloti*), at embryonic day 35 (E35), astroblasts and immature astrocytes have been identified in the midbrain by their structural properties, such as the presence of gliofilaments and dense glycogen granules. Astrocytes have been also found in the white matter of this lizard species and in grey matter in small numbers (Monzon-Mayor et al., 1990). At E35, Vimentin^+^ RG cells line the ventricles and send fibers that run radially into the cortex and ventral striatum, and throughout the basal nuclei. The endfeet of these fibers contact blood vessels and are intensively stained with Vimentin at this stage, while they are weakly immunoreactive for GFAP; GFAP immunoreactivity grows in intensity at E40. In the adult, an uneven expression of GFAP is retained in the RG fibers in the basal nuclei, cortex, and walls of lateral vessels. However, there are no stellate astrocytes in the cortex or hippocampus, and very few are present in the optic tectum together with predominant RG processes (Yanes et al., 1990). In the jewelled lizard (*Timon Lepidus*), the same three types of glia are present: ependymal cells, RG, and free astrocytes. In the spinal cord, RG surround the ependymal layer, while in white matter, astrocyte morphology is more developed in the ventral vs the dorsal portions. Transitional elements with an intermediate morphology between RG and astrocytes have been documented in both white and grey matter (Bodega et al., 1990), with a simpler morphology than that of mammalian astrocytes. In grey matter, cells similar to mammalian protoplasmic astrocytes are visible, with variable number and orientation of the processes depending on the region (few/short dorsally and numerous/more complex ventrally). Elements similar to fibrous astrocytes with long and numerous processes have also been documented in white matter. In general, perineuronal astrocytes are more abundant than in other lizard species and are often associated with blood vessels (Bodega et al., 1990).

In the Japanese striped snake (*Elaphe quadrivirgata*), there are several GFAP^+^ RG with radial processes and a very small number of stellate-shaped astrocytes only in the optic tract and molecular layer of the gyrus dentatus in the hippocampus (Onteniente, Kimura, and Maeda 1983). In the boa (*Epicrates maurus*) and python (*Python regius*), GFAP expression is null in the most rostral telencephalon and the DVR and is high in the caudo-medial pallium, septum, striatum, and preoptic hypothalamus (where the radial pattern becomes absent). Free stellate astrocytes are visible in the spinal cord of the python (Lõrincz and Kálmán 2020) (Figure 3G). The corn snake (*Pantherophis guttatus*) shows cells with clear astrocyte morphology in the telencephalon (i.e., in the septum), in the mesencephalon (i.e., in the tecum, tegmentum, and isthmus), and in the spinal cord. In this species, GFAP expression is detectable even in more rostral brain parts (Lõrincz and Kálmán 2020) (Figure 3H,I). In the pit viper (*Bothrops jararaca*), ependymal cells are present in a single layer around ventricles, the cerebral aqueduct, and the central canal of the spinal cord, with cilia arising from basal bodies. Astrocytes represent a homogeneous cell population in terms of density, but one that is more irregular in terms of cell body shape, cytoplasmic organelle distribution, and nuclei shape. Lastly, there are no distinguishable protoplasmic or fibrous astrocytes in snakes (Bondan et al., 2015).

All turtle species investigated in the literature thus far present an abundance of RG cells but no stellate astrocytes. In particular, in the adult turtle (*Mauremys japonica, Mauremys sinensis,* and *Pelomedusa subrufa*) GFAP^+^ RG processes travel from the ventricle to the pia, with few branches and strict contact to blood vessels (Onteniente, Kimura, and Maeda 1983). Here, the GFAP expression distribution is quite homogeneous, without lighter areas like those found in lizards (Lõrincz and Kálmán 2020).

Interestingly, the reptiles belonging to the clade of Archosaurs—a group that includes crocodilians and birds—show the highest density and degree of regional adaptation for stellate astrocytes among reptiles, although ependymoglia and RG are still the predominant types of GFAP^+^ glia. In the common caiman (*Caiman crocodilus*), the presence of GFAP^+^ astrocytes is highly heterogeneous and does not correlate with brain wall thickness. Here, the astrocytes are more numerous than in other reptiles, but less numerous than in birds. Astrocytes are visible in the middle and posterior parts of the telencephalon and in the striatum. They are also present in the diencephalon, in the mesencephalon, and in the spinal cord, where they are intermingled with RG fibers. In the caiman’s cerebellum, GFAP^+^ RG fibers are numerous and so are the astrocytes between them, similar to the Bergmann glia of mammals and birds (Kálmán and Pritz 2001). In the Cuvier’s dwarf caiman (*Paleosuchus palpebrosus*), RG processes are intermingled with non-radial process like in the common caiman; however, thick radial astroglial processes are not present in this species (Kálmán and Pritz 2001).

From a functional perspective, comparative studies have been done on astrocyte activation during wound healing in the gecko (*Gekko japonicus*) vs the rat. The gecko, like other reptiles, is an interesting animal to study in terms of regeneration and wound healing. Astrocyte activation is attenuated in gecko vs rats, resulting in a more efficient wound healing process. This attenuation may be due to different secreted factors, by comparing RNA sequencing (RNA-seq) data from adult gecko, adult rat, and embryonic rat (E18) astrocytes in wound healing models. The different astrocyte responses in different species may have an endogenous origin. Interestingly, RNA-seq data revealed that adult gecko astrocytes express genes similar to those of E18 rat astrocytes (i.e., genes involved in migration and proliferation), pointing to a conservation of astrocyte response to injury between reptiles and mammals (Gu et al., 2015). Moreover, gecko adult astrocytes retain an immature phenotype, resembling rat embryonic astrocytes, because of sustained Vav1 expression (Du et al., 2021).

### Astroglia in birds

Astrocyte morphology and functions in birds are similar to those of mammals. In the chaffinch (*Fringilla coelebs*), few primitive ependymal cells and a network of both protoplasmic and fibrous astrocytes have been observed. Astrocytes send processes that contact blood vessels with typical mammalian-like vascular endfeet, and they wrap neuronal synapses, suggesting a role in synapse formation and functions (Achúcarro N. 1915). In the chicken (*Gallus domesticus*), astroglial cells with 4–8 processes and a round/oval soma had already been found in both the dorsolateral and the ventromedial portion of the pallium in 1966, with processes encompassing the neuronal surface and in close association with blood vessels (Bairati and Maccagnani 1950; King 1966). Finally, in the finch (*Lonchura striata*), a variable number of GFAP^+^ astrocytes have been observed in white and grey matter, with few perivascular astrocytes close to the ependymal layers (Bairati and Maccagnani 1950). However, to the best of my knowledge, no studies are available to show the distribution of astrocyte populations different from the GFAP^+^ population in birds.

### Astroglia in mammals

Astrocytes as we know them today have been mostly investigated in mammals, and more specifically in the mouse, in humans, and in non-human primates. Although scarce, studies in different mammalian species point to great astrocyte heterogeneity across mammals, with specific astrocyte morphology, density, and functions in primates (Oberheim et al., 2009; Verkhratsky et al., 2018; Falcone et al., 2019; 2021).

A compelling electron microscopy study on monotremes (the most ancient mammals) showed that there are strong differences between the monotremes and therian mammals in terms of glial structure and function (Lambeth and Blunt 1975). The authors showed clear differences between neuronal and glial cells, with the latter being smaller size and having denser cytoplasmic and nucleoplasmic matrices. The microtubules contained in such glial cells are neither arranged circumferentially (as in therian oligodendrocytes) nor associated with filaments (as in therian astrocytes). Only one type of macroglia was found in the platypus (*Ornithorhynchus anatinus*) and in the echidna (*Tachyglossus aculeatus*) immature brain, with a lighter and a darker variant in the echidna (based on electron microscopy observations). However, this glial cell type morphologically resembles neither the astrocytes nor the oligodendrocytes of therian mammals, yet has the potential to exert the functions of both therian astrocytes and oligodendrocytes (Lambeth and Blunt 1975). Unfortunately, no recent studies are available to offer more details about glial cells in monotremes.

Among marsupials, the opossum (*Didelphis virginiana*) presents astrocytes and transitional glia elements, while it lacks adult ependymal cells with processes projecting from the ventricle, as we observe in other non-mammalian species. Opossum astrocytes have round, oval, polygonal cell bodies with 4–12 fine and thicker processes that often contact blood vessels or end around neurons or on other glial cells. They are present in the hippocampus, often with processes running parallel to the apical dendrites of the pyramidal cells. In the neocortex, astrocytes sparsely populate all cortical layers and are denser than in the archipallium. Cortical astrocytes’ main processes are thick, with secondary branches, and their endfeet contact blood vessels in the parenchyma and close to the pial surface. In caudate and lentiform nuclei, there are glial elements similar to those of the cell types found in the neopallium, with some thin processes contacting neuronal fibers (King 1966).

In cattle (*Bos taurus*) and horse (*Equus caballus*), a wide network of protoplasmic astrocytes and perivascular astrocytes have been observed (Bairati and Tripoli 1954). In the hippocampus, astrocyte processes contact pyramidal neurons, and in the cortex, astrocyte endfeet on the vasculature are present. Older studies also reported transitional forms of astrocytes, with a morphology intermediate between protoplasmic and fibrous astrocytes, and between protoplasmic astrocytes and oligodendrocytes (Contu P. 1954).

Astrocyte spatial organization has been studied in depth in the ferret (*Mustela putorius*) visual cortex. These astrocytes are twice as large as mouse/rat cortical and hippocampal astrocytes, but smaller than those in humans. Intriguingly, the classical astrocyte tiling model may not apply to the ferret visual cortex, where there is a large overlap of the processes of neighboring astrocytes (López-Hidalgo, Hoover, and Schummers 2016).

Interestingly, Bergmann glia, a specialized type of unipolar astrocytes derived from RG and intimately associated with Purkinje cells in the cerebellum, can be found in all mammals.

Astrocyte heterogeneity from a morphological, spatial, molecular, and functional perspective has been thoroughly investigated in the mouse (*Mus musculus*), thanks to state-of-the art technologies (Matyash and Kettenmann 2010; Bayraktar et al., 2020; Westergard and Rothstein 2020). In terms of regional specialization, protoplasmic astrocytes in the cortex and hippocampus possess more branches than those in the hypothalamus and in other subcortical regions, which allows their anatomical domain size to be larger. Moreover, a transcriptomic analysis revealed region-specific and cortical layer-specific gene expression in astrocytes too, similar to what happens for neurons (Bayraktar et al., 2020). Finally, physiological Ca^2+^ activity is also region-dependent in the mouse (Yang and Jackson 2019). While this high degree of specialization and heterogeneity has been confirmed in humans, further molecular studies will be necessary to investigate astrocyte heterogeneity in a larger cohort of different mammalian orders.

### Astroglia in human and non-human primates

Recently, increasing attention has been paid to astrocytes in the brains of human and non-human primates not only due to astrocytes’ relevance in human pathology research but also due to their extraordinary complexity and heterogeneity, as well as their potential role in the evolution of advanced cognitive functions. An increase in astrocyte morphological complexity in primates vs mouse was already evident in old comparative studies of astrocytes. King et al. demonstrated that Rhesus macaque astrocytes are increased in number, length, and thickness of the processes, with an stronger glia-vascular and glia-neuronal relationship (King 1966).

**FIGURE 4:**
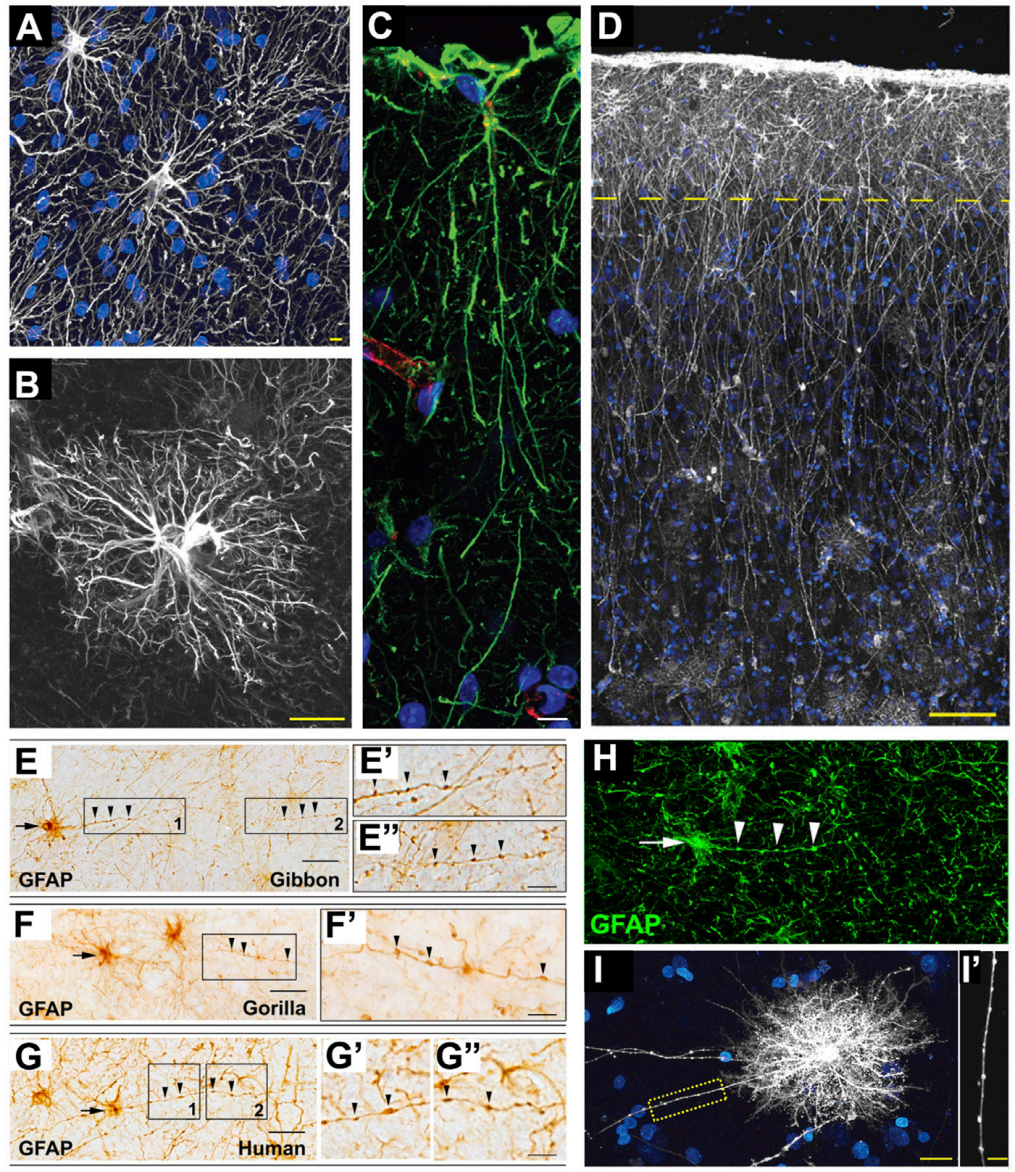
Astrocytes in hominid brains. **(A)** Adapted from Oberheim et al., 2009, Figure 4B. Typical human protoplasmic astrocyte. White: GFAP; blue: DAPI. Scale bar: 20 µm. **(B)** Adapted from Oberheim et al., 2009, Figure 7B. Human fibrous astrocytes in white matter. Grey: GFAP. Scale bar: 10 µm. **(C)** Example of ILA in the rhesus macaque dorsofrontal cortex. Green: GFAP; Red: Lectin; Blue: DAPI. Scale bar: 20 µm. **(D)** Adapted from Oberheim et al., 2009, Figure 3A, showing ILA palisade. Pial surface and layers one to two of human cortex. Dashed yellow line indicates border between layer 1 and 2. White: GFAP; Blue: DAPI. Scale bar, 100 µm. **(E–E”)** Adapted from Falcone et al., 2021, Figure 2A. GFAP + VP-A in frontal cortex of a gibbon. Scale bar: 30 μm. **(E′,E″)** Higher magnification of 1 and 2 in E, respectively. Scale bar = 10 μm. **(F,F′)** Adapted from Falcone et al., 2021, Figure 2C. GFAP + VP-A in frontal cortex of a gorilla. Scale bar = 30 μm. **(F′)** Higher magnification of squared area in **(F)**. Scale bar: 10 μm. **(G–G″)** Adapted from Falcone et al., 2021, Figure 2D. GFAP^+^ VP-A in the human frontal cortex. Scale bar = 30 μm. **(G′,G″)** Higher magnification of 1 and 2 in G, respectively. Arrows point to cell somata, arrowheads point to varicosities on VP-A processes. Scale bar: 10 μm. **(H)** Adapted from Falcone et al., 2021, Figure 3E. VP-A in human frontal cortex. Green = GFAP; Blue = DAPI. Scale bar = 20 μm. Arrows point to cell somata, arrowheads point to varicosities on the VP-A processes. **(I,I′)** Adapted from Oberheim et al., 2009, Figure 2B. Diolistic labeling (white) of a VP-A whose long process terminates in the neuropil. Blue = sytox. Scale bar: 20 µm. **(I′)** High-power image of the yellow box in I highlighting the varicosities seen along the processes. Scale bar, 10 µm.

While protoplasmic and fibrous astrocytes can be found in all mammalian species (Figures 4A,B), there are two types of astrocytes that reach a high degree of specialization in the primate cerebral cortex: interlaminar astrocytes (ILAs) and varicose-projection astrocytes (VP-As).

ILAs have their cell body in cortical layer I, very close to the pia, and have long, GFAP^+^ interlaminar processes that travel perpendicular to the pia toward deeper cortical layers, reaching layer IV in humans (Figure 4C). *Pial* ILAs are strictly associated with the pial surface, while subpial ILAs have their somata in upper layer I but are not attached to the pia. ILAs were considered a primate-specific cell type for long time (Jorge A. Colombo and Reisin 2004). They were first described by Andriezen, who initially named them *caudate neuroglial fiber cells* in 1893 (Andriezen 1893). Ramón y Cajal and Retzius included ILAs in their drawings of the human cerebral cortex. Nearly 100 years after, Colombo thoroughly described these unique cells in the brains of several species of primates (i.e., *Sapajus apella* and *Saimiri sciureus,* among others), including human (J. A. Colombo et al., 1995; J. A. Colombo 1996; J. A. Colombo et al., 1997; 2000; Jorge A. Colombo and Reisin 2004). He was the first to use the term *interlaminar astroglia* for this astrocyte subtype. With these studies, his group suggested that the long ILA processes represent a predominant feature of the postnatal primate cerebral cortex and form “palisades” due to their abundance and densely-packed distribution. Colombo’s group hypothesized a potential role for ILAs in the columnar organization of the primate cerebral cortex; however; ILA functions are still largely unknown (Jorge A. Colombo and Reisin 2004). ILAs, with diverse morphologies, have more recently been found to be present in all mammals, suggesting a very ancient origin for ILAs during mammalian evolution. In fact, in a study of 46 mammalian species (22 of which were primates), some mammals were found to have a rudimentary form of ILAs (*rudimentary ILAs,* observed in marsupials, Xenarthra, rodents, Scandentia, Chiroptera, Carnivora, and Artiodactyla), with processes not crossing the layer I–II boundary, while others displayed the typical form of ILAs (*typical ILAs*, observed in primates, Hyracoidea, and Proboscidea), with proper inter-layer processes (Falcone et al., 2019). However, ILAs have specific features in primates that are not present in non-primates: higher density, higher morphological complexity (i.e., ILA processes are more numerous, longer, and more branched), a specific developmental trajectory, and specific molecular markers (i.e., S100b, HOPX, and CRYAB, together with the astrocyte markers Vimentin, Glast, and Aqp4) (Falcone et al., 2019; 2020). Interestingly, among primates, the ILAs of the great apes have greater morphological complexity than other primates. ILAs appear during prenatal development, putatively originate from local RG cells, proliferate at their final destination close to the pia, but reach their final maturation and morphological complexity only after birth in both macaque and human (Falcone et al., 2020). Primate- and great ape-specific ILA features point to specific functions that ILAs might exert in primates’ brain complexity and cognition; however, further studies are necessary to elucidate those functions.

VP-As are another fascinating type of GFAP^+^ astrocyte present in deeper layers of the cerebral cortex and in white matter; they are characterized by bushy short processes and one to five long processes—spanning all directions—with prominent, evenly spaced varicosities (Figures 4D–H). They were first described by Dr. Needergard’s group, which showed their morphology and presence in several human cortex specimens and one chimpanzee cortex specimen (Oberheim et al., 2006; 2009). VP-As have since been referred to as “human-specific” astrocytes (Verkhratsky et al., 2018); however, a more recent study has described their presence in five different species of great apes, as well (i.e., gorilla, *Gorilla*; bonobo, *Pan paniscus*; chimpanzee, *Pan troglodytes*; orangutan, *Pongo pygmaeus*; and gibbon, *Hylobates muelleri*) (Falcone et al., 2021), with comparable morphology in terms of soma shape and size, process length, and density of varicosities. The authors also screened several other species of primates from Old and New World monkeys, as well as prosimians, but they did not detect VP-As in any of these non-hominoid species. Furthermore, VP-As were not present in other mammals with large brains either (i.e., African elephant, *Loxodonta africana*; minke whale, *Balaenoptera acutorostrata*; or giraffe, *Giraffa camelopardalis*), indicating that they are not a feature of larger brains per se, but seem to occur only in human and apes, probably due to mechanisms that evolved in a common hominoid ancestor. Intriguingly, VP-As were not present in all human and ape individuals analyzed, leading to the hypothesis that they might represent a modified form of astrocytes undergoing a morphological change in response to specific brain conditions (Falcone et al., 2021). However, more studies are needed to identify the specific functions of VP-As.

Thanks to state-of-the-art single-cell RNA-seq technology, recent findings have shed light on the species-specific molecular profile of astrocytes. Human protoplasmic and fibrous astrocytes not only are morphologically different from their mouse counterparts (i.e., they are bigger and more complex) but also show a species-dependent transcriptomic profile that is intrinsically programmed (Li et al., 2021). In fact, human astrocytes display a certain degree of conserved gene expression compared to mouse, but also thousands of differentially expressed genes. In particular, human astrocytes show higher expression of genes involved in defense response to stress and genes linked with the extracellular space and secreted factors, while mouse astrocytes exhibit higher expression in genes involved in the regulation of metabolism and mitochondrial respiration (Li et al., 2021). Moreover, an RNA-seq analysis of induced pluripotent stem cell-derived astrocytes from human and chimpanzee showed inter-species differential gene expression in astrocytes, with notable differences in cellular respiration, glucose and lactate transmembrane transport, and pyruvate utilization, suggesting higher metabolic capabilities of human vs chimpanzee astrocytes (Zintel et al., 2020).

## Discussion

Astrocytes are highly heterogeneous across different CNS regions, developmental stages, and species (Oberheim et al., 2009; Matyash and Kettenmann 2010; Oberheim, Goldman, and Nedergaard 2012; Verkhratsky et al., 2018; Yang and Jackson 2019; Bayraktar et al., 2020; Westergard and Rothstein 2020; Falcone et al., 2021). Findings related to the distribution and presence of astrocyte and astrocyte-like cells are scattered across several species of invertebrates and vertebrates (Supplementary Table S1), and, most of the times, relie on investigating their morphology, which is often revealed by immunohistochemistry against the GFAP marker. Ependymal cells and RG are currently hypothesized to be the predecessors of astrocytes in non-mammalian species. GFAP^+^ ependymal cells are abundant in fish and amphibians, have variable distribution in reptiles, and are very scarce in birds and mammals. RG cells are present in all vertebrates, but with variable distribution: they are still the predominant type of astroglia-like cells in fish and amphibians, and in reptiles, they are retained in the adult and exert functions similar to those of mammalian astrocytes. Proper stellate astrocytes were historically first identified in mammals (where fibrous and protoplasmic astrocytes were first distinguished), but have been also observed in birds and reptiles, where they contact blood vessels and synapses and begin to show features typical of astrocytes, like those we see in mammals (Suárez et al., 1995). Interestingly, in reptiles, intermediate forms between RG cells and astrocytes can be found; however, in adult mammals, most RG cells are replaced by astrocytes that retain some plasticity and their ability to proliferate under specific conditions. GFAP^+^ astrocytes are found in lizards, snakes, and caimans, often intermingled within RG fibers, while being absent in turtles (Onteniente, Kimura, and Maeda 1983). The appearance of astrocytes in reptiles is concomitant with the reduction of ependymal cells and RG. This phenomenon points to reptiles being a key group in the phylogenetic evolution of astrocytes. Moreover, GFAP-free areas and the presence and diversity of astrocytes increased during evolution (Lõrincz and Kálmán 2020). The mixed presence of RG and astrocytes in reptiles suggests that the appearance of astrocytes anticipated and maybe contributed to increases in brain size and complexity. Astrocytes, in contrast to RG cells, have the advantage of forming a dynamic network that can provide local adaptability. In general, findings regarding astrocytes across evolution show a trend of the progressive regional adaptation of the glial structure, resulting in extraordinary astrocyte heterogeneity in primates and more specifically in humans (Kálmán and Pritz 2001).

However, the appearance of astrocytes in evolution has been proposed to be an apomorphic trait, especially when comparing caimans and turtles to phylogenetically related birds (i.e., chicken). For example, the presence of RG in several brain regions, often with an arched course of RG fibers, is a feature shared by caiman, turtles, and chicken. In contrast, GFAP expression is present everywhere in caiman (and astrocytes are intermingled with RG fibers and never predominant), while that expression is absent in turtle and predominant in birds.

We still have a long road ahead in understanding where astrocyte diversity comes from. To go farther down that road, a few new steps must be taken. First, to counteract the limitations linked to the use of GFAP to detect the presence of astrocytes, there is an urgent need to compare the expression of other astrocyte markers and specific GFAP isoforms (e.g. GFAP-α, GFAP-δ) across vertebrates. Moreover, more modern techniques, such as bulk and single-cell RNA profiling and spatial transcriptomics, will tell us more about astrocyte heterogeneity and origin across evolution (Yang and Jackson 2019). Finally, due to the high variability of astrocyte distribution, it is imperative that we investigate not only model organisms but also non-model organisms, so that important details about astrocyte evolution can be reconstructed.

Understanding how astrocytes evolved across vertebrates, and more specifically in mammals, is important information not only for its own sake but also because it can offer insights into primate-specific astrocytic pathologies, which are currently difficult to model in rodents.
