# Supplementary material for: Evolution of astrocytes: From invertebrates to vertebrates
Source: Front Cell Dev Biol. 2022 Aug 15;10:931311. doi: 10.3389/fcell.2022.931311 (PMC9423676; doi:10.3389/fcell.2022.931311)
Supplement: Supplementary file 1 [file Table1.DOCX]

**Table 1. Summary of most important findings related to astrocyte and astrocyte-like cell presence across invertebrates and vertebrates**

| **Animal group/ Subphylum** | **Class** | **Astrocyte presence** |
| --- | --- | --- |
| **Invertebrates** |  | - Acoelomorpha: first glial cells  *- C. elegans* and Anellida: first glial cells with anatomical and functional features of both mammalian astrocytes and oligodendrocytes (e.g. proto-astrocytes)  *- D. melanogaster*: astrocyte-like glia with physiology similar to mammalian astrocytes (e.g. role in synaptogenesis, calcium oscillations) |
| **Vertebrates** | Fish | - Ependymal cells are the prendominant type of glia in several telost species  - D. rerio: RG/astrocyte-like cells with physiology similar to mammalian astrocytes (e.g. similar markers, calcium oscillations, conserved morphogenesis |
|  | Amphibians | - Ependymal cells and RG are the predominant form of glia (smilar function to mammalian astrocytes) |
|  | Reptiles | - Squamata: RG cells are still predominant, but free astrocyte-like cells are visible within RG fibers (similar morphology to mammalian astrocytes)  - Archosaurs: RG cells are still predominant, but highest density of free stellate astrocyte-like cells within Reptiles  - Turtles: no free astrocyte-like cells |
|  | Birds | -Astrocytes with morphology and functions similar to mammalian astrocytes |
|  | Mammals | - RG and ependymal cells are still present but in lower quantity and mostly during development  - Typical astrocytes are present and abundant; protoplasmic and fibrous astrocytes are present in all mammals; ILAs are present in all with different degrees of morphological complexity (rudimentary vs. typical ILAs) |
|  | Mammals (Non-human primates) | - Astrocytes reach the highest degree of morphological complexity (both in protoplasmic, fibrous and ILAs) within mammals |
|  | Mammals (Humans and Great apes) | - Presence of VPAs, a great-ape specific type of astrocyte |
|  | | |
